# Cost-effectiveness analysis of early antihypertensive treatment for hypertension detected at health screening

**DOI:** 10.1038/s41440-026-02776-5

**Published:** 2026-08-19

**Authors:** Hiromasa Ito, Tomohisa Seki, Toru Takiguchi, Yu Akagi, Kazumi Kubota, Kana Miyake, Masafumi Okada, Kazuhiko Ohe, Yoshimasa Kawazoe

**Affiliations:** 1 Department of Cardiology and Nephrology, Mie University Graduate School of Medicine, Mie, Japan; 2 Department of Healthcare Information Management, The University of Tokyo Hospital, Tokyo, Japan; 3 Department of Applied Health Informatics, Graduate School of Medicine, Dentistry and Pharmaceutical Sciences, Okayama University, Okayama, Japan; 4 Department of Emergency and Critical Care Medicine, Nippon Medical School, Tokyo, Japan; 5 Department of Biomedical Informatics, Graduate School of Medicine, The University of Tokyo, Tokyo, Japan; 6 Artificial Intelligence and Digital Twin in Healthcare, Graduate School of Medicine, The University of Tokyo, Tokyo, Japan

**Keywords:** Implemental Hypertension, Antihypertensive therapy, Cost-effectiveness analysis, Health screening, Markov model

## Abstract

Background: Delayed initiation of antihypertensive therapy after the initial identification of hypertension during health checkups has been linked to an increased risk of cardiovascular disease. However, the long-term economic implications of this practice are not well understood. This study aimed to analyze the cost-effectiveness of early antihypertensive treatment. Methods: A Markov cohort model with four health states (baseline hypertension, acute cardiovascular disease (CVD), post-CVD, and death) was developed to project long-term outcomes for adults with screening-detected hypertension using data from the Japan Medical Data Center database, recorded January 2005–April 2021. Age-specific transition probabilities, CVD-related costs, and mortality were derived from a previously published cohort. Quality-adjusted life-years (QALYs) were estimated using utility values from prior literature. Early (<1 year) versus delayed (≥1 year) treatment initiation were compared across four age groups over a 20-year horizon from the healthcare payer perspective, applying a 2% annual discount rate. Deterministic and probabilistic sensitivity analyses assessed parameter uncertainty, and cost-effectiveness was evaluated against a willingness-to-pay threshold of ¥5 million per QALY. Results: In adults in their 40 s, early initiation yielded 13.52 QALYs and lifetime costs of ¥3,991,757, compared with 12.85 QALYs and ¥3,976,709 with delayed initiation, resulting in an incremental cost-effectiveness ratio of ¥22,489 per QALY. Early initiation remained highly cost-effective across all age groups, and probabilistic analyses demonstrated a high likelihood of cost-effectiveness. Conclusions: These findings support encouraging healthcare payers to use screening results proactively to promote early hypertension management and strengthen follow-up systems that reduce delays between detection and appropriate treatment.

**Figure:**
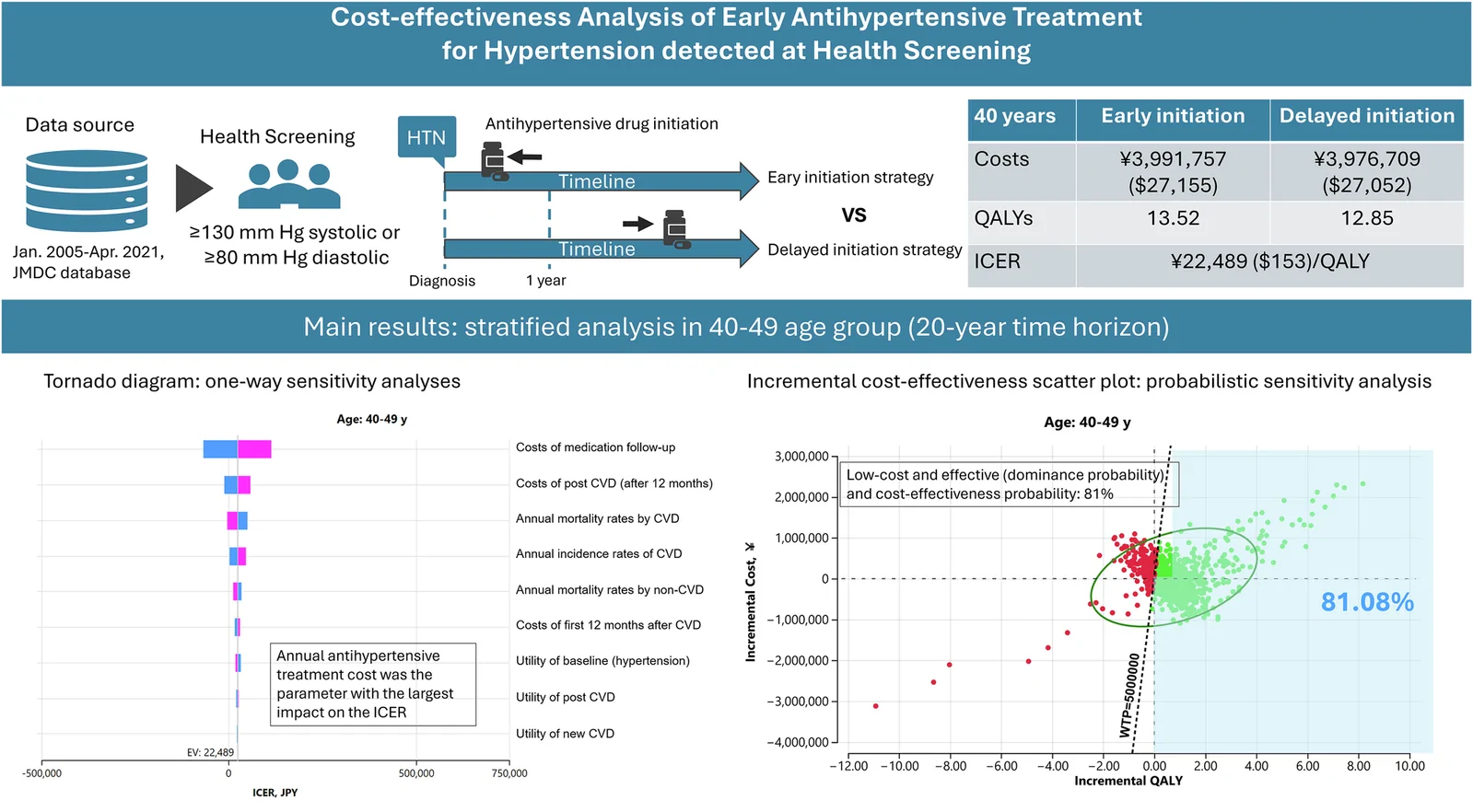

## Introduction

Hypertension is a major global health concern and is widely acknowledged as a leading contributor to the development of cardiovascular and cerebrovascular disorders, such as stroke, myocardial infarction, heart failure, and chronic kidney disease [1–4]. In many Asian countries, including Japan, its prevalence continues to rise, driven largely by population aging and lifestyle transitions, making early detection and appropriate intervention increasingly urgent [5–10].

Cardiovascular disease (CVD) imposes a substantial and growing economic burden worldwide. Healthcare expenditures are increasing due to aging populations and multimorbidity, along with the rising costs of medical devices and pharmaceuticals [1, 11, 12]. In Japan, CVD is one of the largest components of national medical spending. Hypertension-related costs, including outpatient care, hospitalization, and medications, continue to rise [13, 14]. These trends underscore the need to identify strategies that can prevent cardiovascular events while ensuring the sustainability of healthcare expenditures.

From a clinical and policy perspective, growing attention has been directed toward optimizing the initiation and intensity of antihypertensive therapy, with the goal of achieving both improved patient outcomes and cost-effective care [15–18]. In previous studies, we reported that delaying the initiation of antihypertensive medication by more than one year following the detection of hypertension during health checkups significantly increased the incidence of cardiovascular events in individuals aged 40 years and over [19]. However, the cost-effectiveness of early initiation of antihypertensive treatment remains unclear across different age groups at the time of hypertension diagnosis. This study aimed to evaluate whether early initiation of antihypertensive medication for hypertension detected during health checkups is cost-effective across different age groups using a Japan health checkup database.

Point of view

- Clinical relevance:
  Initiating antihypertensive therapy within 1 year of screening-detected hypertension is highly cost-effective across all adult age groups, supporting stronger follow-up systems after health checkups.
- Future direction:
  Prospective and implementation studies are needed to test interventions that shorten the interval between screening detection and treatment initiation in real-world practice.
- Consideration for the Asian population:
  Widespread health checkup infrastructure across Asia offers a practical platform for closing the detection-to-treatment gap in hypertension management region-wide.

## Methods

An economic model to evaluate the long-term costs and effectiveness of the early initiation of antihypertensive therapy for hypertension detected through screening was developed. The model estimated quality-adjusted life years (QALYs) and lifetime medical costs under two strategies: early initiation of antihypertensive medication within one year after detection of hypertension, and delayed initiation, one year or more after detection. A lifetime horizon with a one-year cycle length was used. The analysis was conducted from the perspective of a public healthcare payer in Japan. In Japan’s universal health insurance system, healthcare payers, including employer-based and community-based insurers, are responsible not only for financing medical care, but also for administering population-wide health screening programs. Therefore, the timing of follow-up and treatment after screening-detected hypertension directly affects payer expenditures and health management outcomes.

The model parameters were based on findings from a previous cohort study [19] and were supplemented with data from existing studies. The analysis was performed according to the Consolidated Health Economic Evaluation Reporting Standards (CHEERS) statement [20]. This study conformed to the principles of the Declaration of Helsinki and was approved by the Ethics Committee of the University of Tokyo Hospital (Approval No. 2024278NIe). This study used anonymized data, and therefore, the requirement for obtaining informed consent from participants was waived.

### Model description

**Fig. 1:**
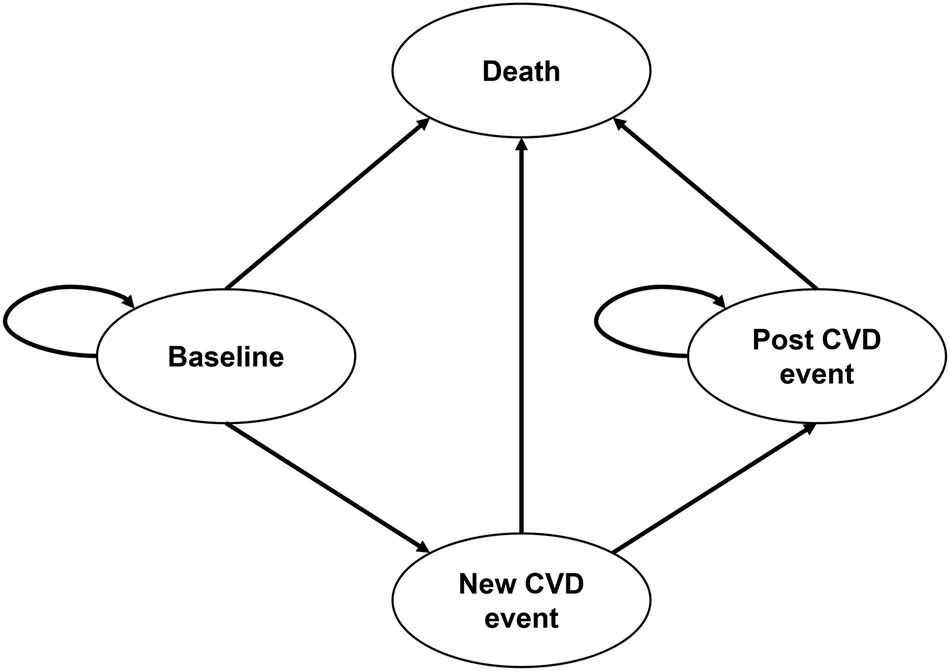
Markov model of early antihypertensive treatment. Each yearly cycle allows transitions among four health states: baseline, new cardiovascular disease (CVD) event, post-CVD event, and death, following the directions indicated by the arrows

A model-based, cost-effectiveness analysis was conducted to evaluate the long-term clinical and economic impact of early initiation of antihypertensive therapy for hypertension detected at health checkups. A state-transition Markov cohort model with an annual cycle length was constructed (Fig. 1). The model included four mutually exclusive health states: baseline with hypertension, new CVD event, post CVD event, and death.

The composite cardiovascular outcome included acute coronary syndrome, heart failure, and stroke requiring hospitalization. All individuals entered the model in the baseline hypertension state. In each annual cycle, they either remained in the baseline state if no CVD event or death occurred, moved from baseline to the new CVD state when they experienced a first cardiovascular event, or died from cardiovascular or non-cardiovascular causes and moved to the death state. Individuals in the new CVD state subsequently moved to the post CVD state if they survived the first year after the event, or moved to the death state if they died during that year.

### Age stratification and time horizon

Because age at health checkup and hypertension detection can substantially affect both baseline risk and the impact of treatment timing, four separate age scenarios based on age at screening were evaluated: 30–39 years, 40–49 years, 50–59 years, and ≥60 years.

The main analysis used a 20-year time horizon. For the scenario analysis, we examined time horizons of 30, 40, and 50 years to evaluate the long-term benefits of reaching 80-89 years for each age group.

### Population and intervention

The modeled population was based on participants in our previous cohort study [19]. Data were recorded from the Japan Medical Data Center (JMDC) database between January 1, 2005, and April 30, 2021. The JMDC database is a commercially available, anonymized, health insurance claims database from Japan. Widely used in real-world studies, it includes longitudinal health checkup data collected from employees and their dependents across numerous companies nationwide [21–26].

In the prior cohort, individuals with hypertension who were first detected during periodic health checkups were identified and followed longitudinally. Adults aged ≥20 years were stratified into five age groups. However, individuals in their 20 s represented only 0.66% of the cohort, which resulted in unstable estimates in the simulation. Thus, the present model included four age groups (30 s, 40 s, 50 s, and ≥60 s) and excluded those in their 20 s. In the previous cohort, the time to treatment initiation (TTI) after hypertension was identified at health screening was classified into three categories: TTI < 1 year, TTI 1- <2 years, and TTI ≥ 2 years. For the present study, these categories were consolidated into two clinically meaningful strategies to enhance interpretability and simplify the model. The early initiation strategy was defined as starting antihypertensive therapy within one year of hypertension detection. The delayed initiation strategy was defined as initiating therapy one or more years after detection. It was assumed that treatment patterns after initiation would be similar between strategies. Follow-up for each individual continued until whichever occurred first: a cardiovascular event, death, disenrollment from the insurer, or the end of the database period; this rule was applied identically in both TTI groups, with no additional study-defined truncation. Annual event probabilities were estimated using follow-up time starting from treatment initiation rather than from the index date, so that the pre-treatment interval, which differs in length between the two groups by definition, did not contribute person-time to either group. Summary characteristics of the underlying cohort used for parameter estimation, including the distribution of follow-up duration by age group and TTI category, are shown in Supplemental Table 1. In a sensitivity analysis, individuals with a confirmed malignancy diagnosis (excluding suspected/rule-out diagnoses, and requiring at least two claims ≥30 days apart within the 1-year period before the index date) or a history of dialysis were excluded, as such conditions may independently influence both treatment initiation and survival; no individuals in this cohort met the dialysis criterion.

### Model input data

The transition probabilities, medical cost data, and parameters used in the model are shown in Table 1. Transition probabilities for the delayed initiation (TTI ≥ 1 year) strategy were calculated as annual event rates for first composite CVD events, CVD-related death, and non-CVD death for each age group. These were converted into transition probabilities for the Markov model. The effectiveness of the early initiation (TTI < 1 year) strategy was modeled as absolute risk reductions (risk differences) in the 1-year incidence of composite CVD events, CVD mortality, and non-CVD mortality, estimated from the prior study comparing TTI < 1 year with TTI ≥ 1 year. For each age group, the event probabilities for the early initiation strategy were derived by subtracting the corresponding risk differences from the baseline probabilities observed in the delayed initiation group. Risk differences were used because they provide an absolute measure of treatment benefit and allowed direct application to age-specific baseline event risks estimated from the cohort. However, since no difference in the risk of CVD mortality was observed in those aged 60 years or older with early treatment, the intervention effect was assumed to be zero. These annual event probabilities reflect an intention-to-treat-type approach: each individual’s event history was attributed to the TTI category established at treatment initiation, regardless of any subsequent treatment discontinuation. Discontinuation was defined as a gap in antihypertensive prescription claims exceeding 90 days within the first year after treatment initiation, distinguished from loss to follow-up due to disenrollment or death using insurer enrollment records.

**Table 1:** Parameters input into the model

	Input (range for sensitivity analysis)			
Parameter	Age: 30–39 y	Age: 40–49 y	Age: 50–59 y	Age: ≥60 y
Probability				
Annual incidence rate of CVD, %	1.240 (1.057–1.424)	1.647 (1.549–1.744)	2.182 (2.060–2.304)	2.585 (2.353–2.817)
Annual mortality rate by CVD, %	5.051 (2.459–7.644)	4.945 (3.889–6.000)	5.237 (4.168–6.306)	4.242 (2.723–5.761)
Annual mortality rate by non-CVD, %	0.182 (0.110–0.253)	0.291 (0.249–0.333)	0.456 (0.398–0.514)	0.663 (0.541–0.785)
Annual cost				
Medication (follow-up cost)	¥225,591 ($1535) (¥212,593–¥238,589)	¥225,766 ($1536) (¥221,824-¥229,708)	¥250,619 ($1705) (246,880-254,358)	¥314,059 ($2136) (305,371–322,747)
First 12 months after CVD	¥830,459 ($5649) (¥717,943–¥942,976)	¥850,974 ($5789) (¥811,035–¥890,912)	¥927,502 ($6310) (¥894,400–¥960,604)	¥959,479 ($6527) (¥906,374–¥1,012,584)
Post CVD (after 12 months)	¥438,636 ($2984) (363,025–514,247)	¥444,923 ($3027) (413,932–475,915)	¥479,000 ($3259) (453,588-504,411)	¥496,997 ($3381) (448,836–545,157)
Effectiveness of intervention (risk difference)				
Composite CVD event	0.634 (0.597–0.672)	0.896 (0.860–0.932)	1.396 (1.321–1.472)	1.578 (1.408–1.749)
CVD death	0.731 (0.554–0.908)	0.990 (0.838–1.142)	0.594 (0.220–0.968)	-
Non-CVD death	0.092 (0.076–0.108)	0.179 (0.161–0.197)	0.309 (0.271–0.346)	0.448 (0.355–0.541)
Utilities				
Baseline (Hypertension)	0.85 (0.70–1.00)	0.85 (0.72–0.98)	0.82 (0.66–0.98)	0.73 (0.65–0.91)
New CVD	0.53 (0.47–0.63)	0.53 (0.46–0.63)	0.54 (0.47–0.64)	0.54 (0.47–0.63)
Post CVD	0.65 (0.53–0.72)	0.65 (0.54–0.72)	0.66 (0.54–0.72)	0.65 (0.53–0.72)

*CVD* cardiovascular disease

All costs were estimated from claims in the JMDC database and expressed in Japanese yen (JPY, ¥). Currency conversion to US dollars (USD, $) was performed using an exchange rate of 1 USD = ¥147 (as of August 2025) for descriptive purposes only; cost-effectiveness analyses were based on JPY. Each cost input was derived by extrapolation: costs were estimated cross-sectionally from claims and then applied as a constant value to every model cycle across the full time horizon for both strategies, rather than tracked separately according to each individual’s specific TTI duration.

Medication costs for hypertension treatment were calculated from annual prescription claims of individuals with hypertension but without CVD events in the cohort used in the previous study. These costs were assumed to reflect routine pharmacological management of hypertension. The cost of antihypertensive medication treatment was ¥225,591 ($1535), ¥225,766 ($1536) for those in their 40 s, ¥250,619 ($1705) for those in their 50 s, and ¥314,059 ($2136) for those aged 60 years and over.

In the early initiation strategy, antihypertensive therapy began within the first year after detection. Thus, an additional year of medication costs was added at model entry. Since some individuals may begin therapy before the one-year mark, a scenario analysis assuming the additional drug cost associated with early initiation corresponded to 0.5 years of treatment rather than a full year was also conducted. Costs of new CVD events were defined as the sum of all medical expenditures recorded in the 12 months starting from the month of the index CVD hospitalization (event month). For example, for a CVD event in April 2020, acute CVD costs were the total costs from April 2019 through March 2020. Post CVD costs were defined as the total medical expenditures during the subsequent 12 months after the acute phase. For example, costs from April 2020 through March 2021 were considered post CVD costs. The cost of death was not modeled separately because it was difficult to distinguish end-of-life expenditures from new and post CVD costs using claims data.

Utility values for QALY calculations are summarized in Table 1. Baseline utility values for hypertensive individuals without prior CVD were derived from studies reporting EQ-5D scores in Japanese general population samples [27]. A utility decrement associated with hypertension of 0.153, based on an EQ-5D study of Japanese adults, was then applied to obtain age-specific baseline utilities for the baseline hypertension state. For the composite CVD outcome, utility values were obtained for acute and chronic phases for acute coronary syndrome, heart failure and stroke from previous research (Supplementary Table 2). The utilities for the new CVD and post CVD states were defined as the probability-weighted average of the utilities for each component. Both costs and QALYs were discounted at an annual rate of 2% in the base-case analysis, in line with recommendations for economic evaluations in Japan [28].

### Scenario analysis and sensitivity analysis

The main analysis calculated the incremental cost-effectiveness ratio (ICER) of the early initiation strategy (TTI < 1 year) compared with the delayed initiation strategy (TTI ≥ 1 year) over a 20-year time horizon for each age group. Scenario analyses were performed to assess: (1) alternative time horizons of 30–50 years instead of 20 years; (2) alternative assumptions about additional antihypertensive treatment costs in the early initiation strategy, in which the extra treatment duration due to early initiation was reduced from 1 year to 0.5 years; (3) a per-protocol sensitivity analysis excluding individuals who discontinued antihypertensive treatment within 1 year of initiation; and (4) a sensitivity analysis excluding individuals with malignancy or a history of dialysis.

To assess parameter uncertainty, both deterministic one-way sensitivity analyses and probabilistic sensitivity analyses (PSA) were conducted. In the one-way sensitivity analyses, transition probabilities, costs, and utilities were varied individually across plausible ranges based on 95% confidence intervals from the cohort data. For the PSA, beta distributions were assigned to clinical event probabilities and utility parameters, and gamma distributions were assigned to cost parameters. A total of 100,000 Monte Carlo simulations were then performed to propagate joint parameter uncertainty. The PSA results are presented as incremental cost-effectiveness scatter plots and cost-effectiveness acceptability curves.

A willingness-to-pay (WTP) threshold of ¥5,000,000 ($34,014) per QALY gained, which is frequently used in economic evaluations and health technology assessments in Japan [29], was adopted.

All statistical analyses for estimating transition probabilities and costs were performed using R version 4.3.3 (R Foundation for Statistical Computing, Vienna, Austria) and Python 3.8.8. The state-transition model and all economic analyses were implemented using TreeAge Pro Healthcare version 2025 R2.0 (TreeAge Software, LLC, Williamstown, MA, USA).

## Results

### Main analysis

In individuals in their 40 s at the time hypertension was first detected, the early initiation strategy yielded 13.52 QALYs and total lifetime costs of ¥3,991,757 per person. In contrast, the delayed initiation strategy produced 12.85 QALYs and ¥3,976,709 in lifetime costs per person. The resulting incremental cost of early initiation was ¥15,048 and the incremental QALYs gained were 0.67, yielding an ICER of ¥22,489 per QALY gained. In all age strata, the ICERs were well below the WTP threshold of ¥5,000,000 per QALY gained (Table 2).

**Table 2:** Cost-effectiveness of early initiation of antihypertensive medication

	Age: 30–39 y		Age: 40–49 y		Age: 50–59 y		Age: ≥60 y	
	TTI < 1 year	TTI ≥ 1 year	TTI < 1 year	TTI ≥ 1 year	TTI < 1 year	TTI ≥ 1 year	TTI < 1 year	TTI ≥ 1 year
20-year time horizon (main)								
Costs	¥3,950,016 ($26,871)	¥3,918,410 ($26,656)	¥3,991,757 ($27,155)	¥3,976,709 ($27,052)	¥4,397,312 ($29,914)	¥4,411,692 ($30,012)	¥5,423,293 ($38,893)	¥5,306,798 ($36,101)
QALYs	13.61	13.16	13.52	12.85	12.99	12.05	11.50	10.66
ICER	¥70,377 ($479)/QALY		¥22,489 ($153)/QALY		-¥15,272 (-$104)/QALY		¥138,998 ($946)/QALY	
30-year time horizon								
Costs	¥5,346,669 ($36,372)	¥5,291,741 ($35,998)	¥5,407,155 ($36,783)	¥5,341,409 ($36,336)	¥5,926,380 ($40,316)	¥5,847,963 ($39,782)		
QALYs	18.30	17.37	18.12	16.76	17.37	15.48		
ICER	¥58,450 ($398)/QALY		¥48,080 ($327)/QALY		¥41,406 ($282)/QALY			
40-year time horizon								
Costs	¥6,451,050 ($43,885)	¥6,324,831 ($43,026)	¥6,521,779 ($44,366)	¥6,339,036 ($43,123)				
QALYs	21.95	20.44	21.66	19.50				
ICER	¥83,480 ($568)/QALY		¥84,736 ($576)/QALY					
50-year time horizon								
Costs	¥7,314,892 ($49,761)	¥7,086,993 ($48,211)						
QALYs	24.77	22.67						
ICER	¥108,355 ($737)/QALY							

*TTI* time to treatment initiation, *QALY* quality-adjusted life-year, *ICER* incremental cost-effectiveness ratio

### Scenario analysis

The results of analyses conducted by changing the time horizon to 30 years, 40 years and 50 years are shown in Table 2. When the incremental healthcare costs associated with earlier initiation of antihypertensive medication were adjusted to 0.5 years (6 months) per year, the ICER was -¥61,859/QALY gained for individuals in their 40 s (Supplementary Table 5). Discontinuation of antihypertensive treatment within 1 year occurred in 17.3% of individuals overall (range 8.9–33.5% across age and TTI strata) and was more frequent in the delayed- than the early-initiation group in every stratum. In the per-protocol sensitivity analysis excluding these individuals (parameters in Supplemental Table 3), the ICER was ¥54,769/QALY gained for individuals in their 40 s (Supplementary Table 6). We additionally performed a sensitivity analysis excluding individuals with malignancy or a history of dialysis (parameters in Supplemental Table 4); 1645 of 522,596 individuals (0.31%) met this exclusion criterion, all through the malignancy criterion. Under this sensitivity analysis, the ICER for early initiation was ¥29,621/QALY gained for individuals in their 40 s (Supplemental Table 7), consistent with the main analysis.

### Sensitivity analyses

**Fig. 2:**
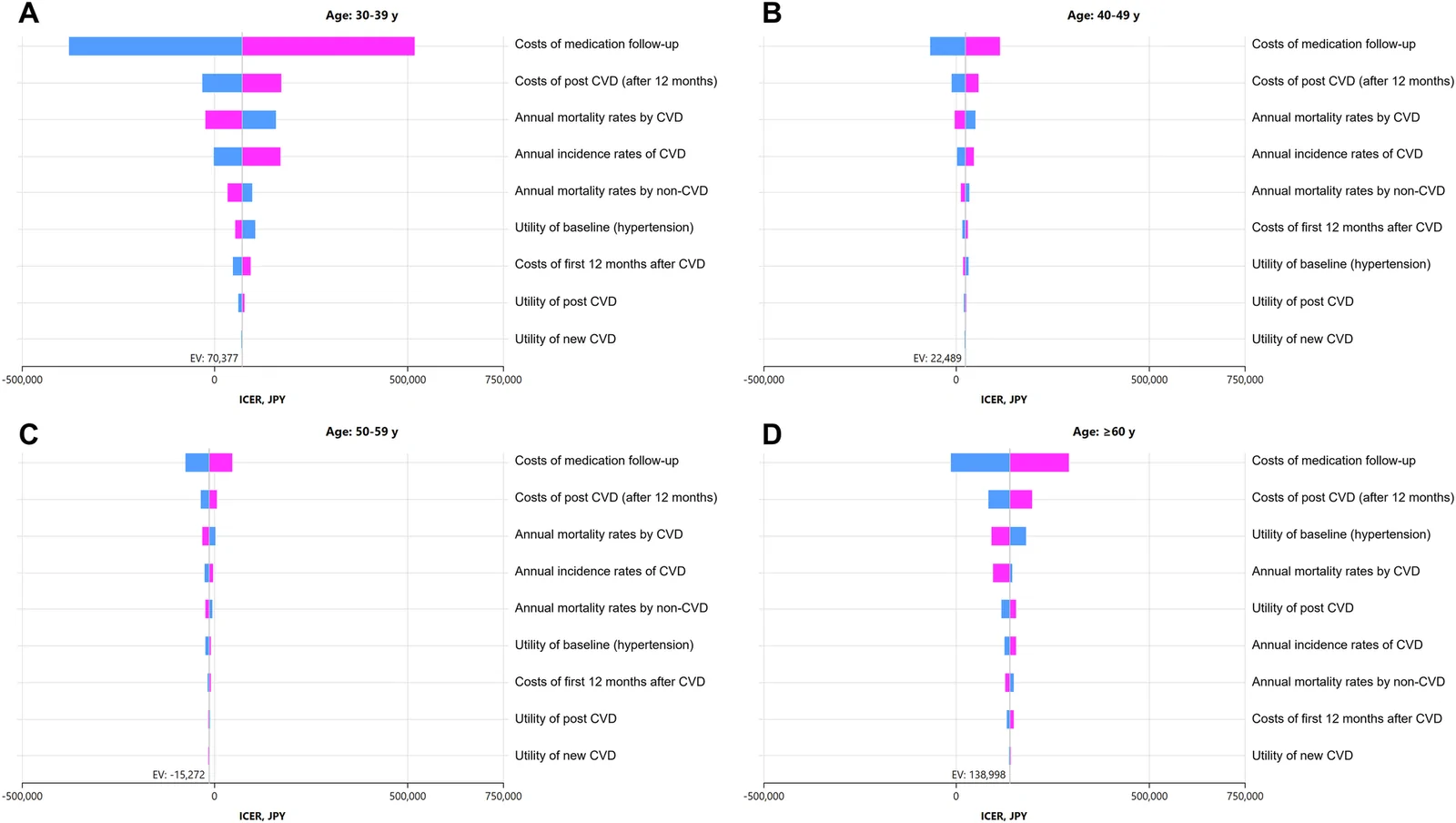
Tornado diagrams from the results of the one-way sensitivity analyses. Each diagram shows how changes in individual model parameters affect the incremental cost-effectiveness ratio (ICER). The horizontal bars represent the range of ICER values produced when each parameter varies across its plausible range. Longer bars indicate greater effects on the model’s outcomes. Parameters are ordered vertically from the most to the least influential. Panels present the results for each age group: **A** 30–39 years; (**B**) 40–49 years; (**C**) 50–59 years; and (**D**) ≥ 60 years. EV: equivalent variation. JPY: Japanese yen

One-way sensitivity analyses are summarized in the tornado diagrams (Fig. 2). Across all age groups, annual antihypertensive treatment cost was the parameter with the largest impact on the ICER. When this parameter was varied across its plausible range, the ICER for early initiation changed substantially but did not exceed ¥520,000 per QALY in any age group. The largest ICER observed in the deterministic analyses was ¥518,588 per QALY in the 30 s age group, still well below the WTP threshold. In any age group, the cost of post-CVD events was the second most influential parameter.

**Fig. 3:**
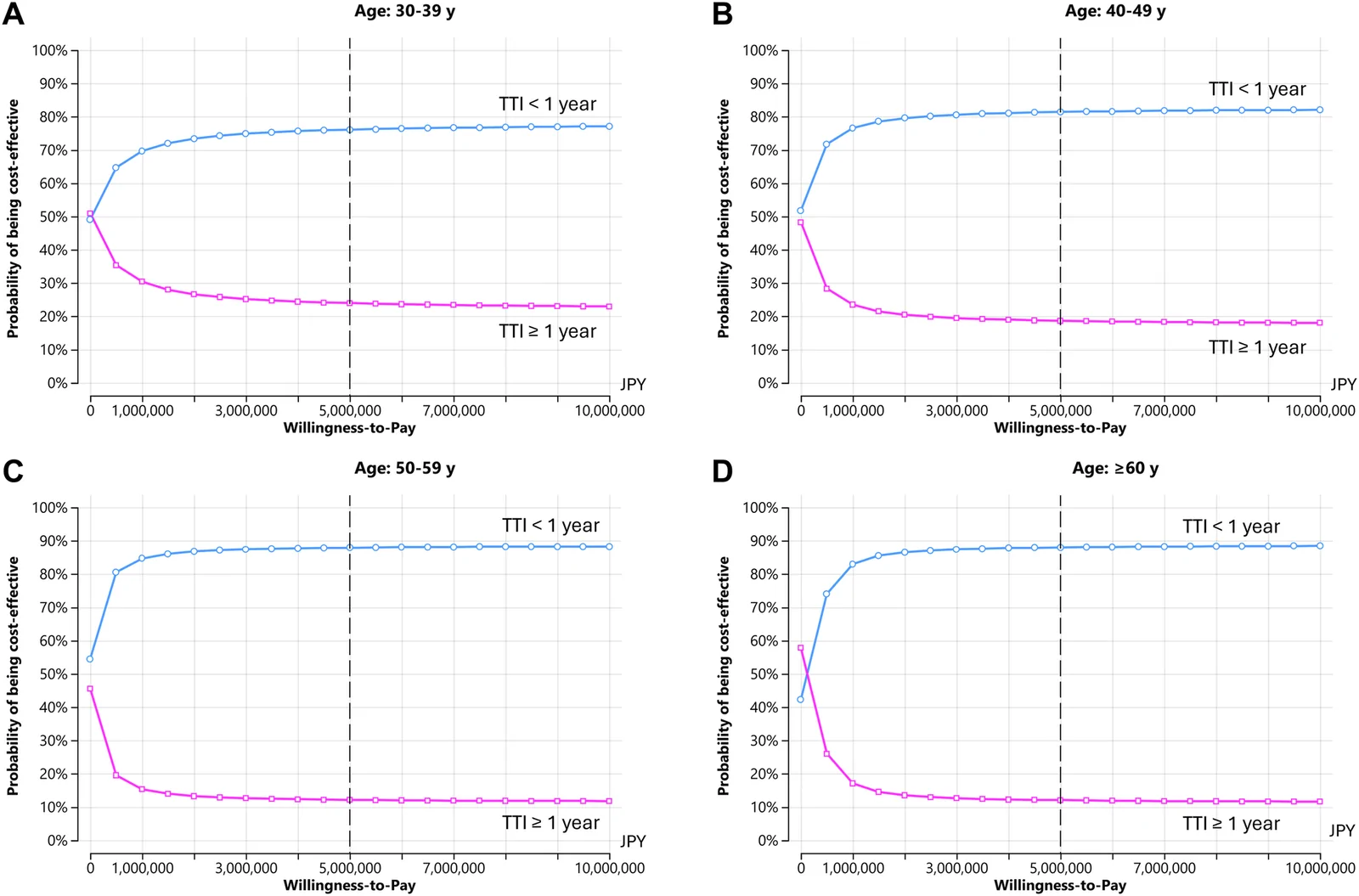
Cost-effectiveness acceptance curve in the early antihypertensive initiation (TTI < 1 year) and the delayed initiation (TTI ≥ 1 year) groups. The curve shows the probability that early antihypertensive treatment is cost-effective at different willingness-to-pay (WTP) levels. The vertical axis represents the probability of cost-effectiveness, whereas the horizontal axis shows the WTP per QALY gained. The two curves intersect at a WTP level below 5 million yen per QALY gained. Panels present the results for each age group: **A** 30–39 years; (**B**) 40–49 years; (**C**) 50–59 years; and (**D**) ≥ 60 years. JPY: Japanese yen

**Fig. 4:**
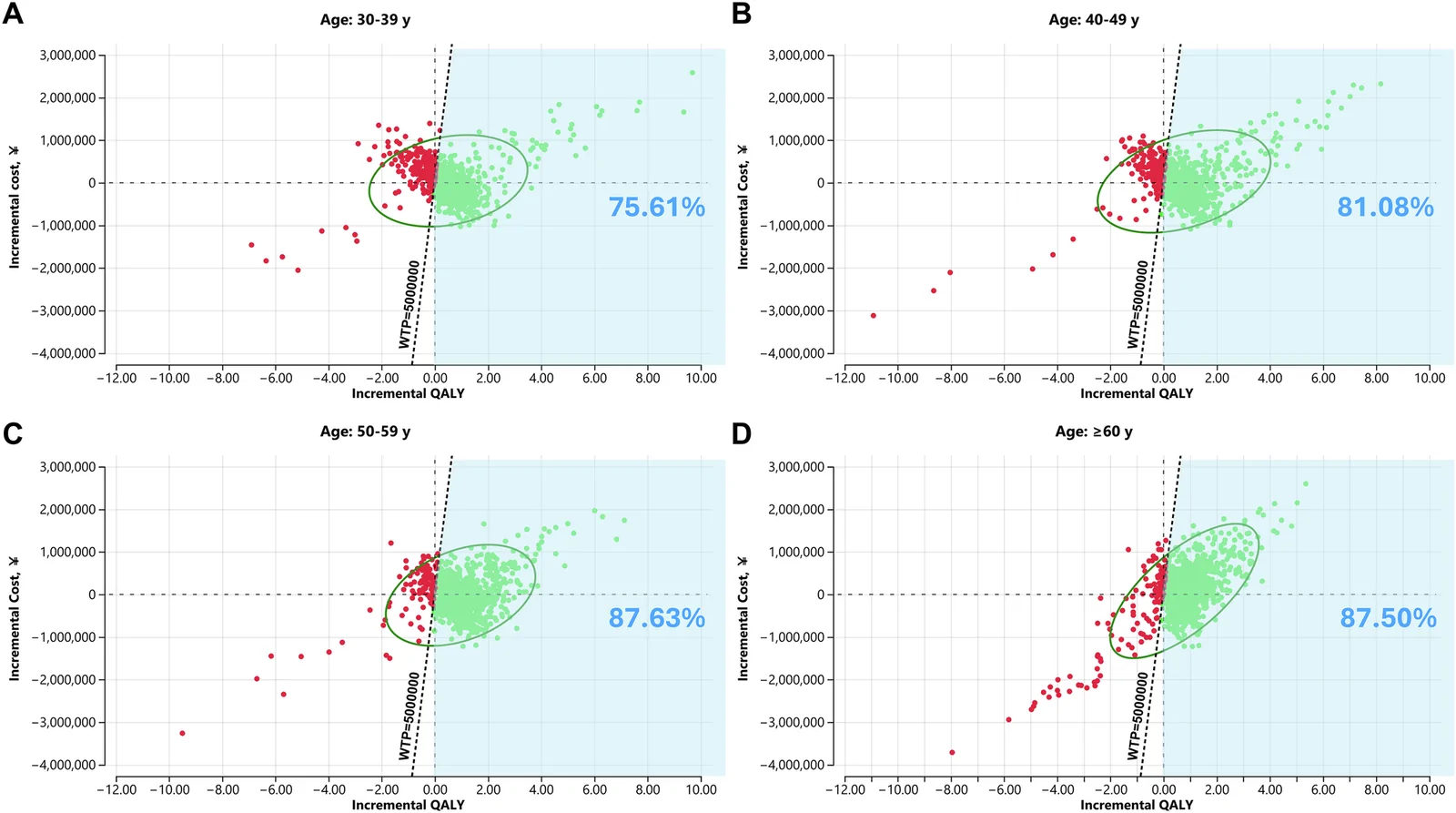
Incremental cost-effectiveness scatter plot. Each point in the panel represents the incremental effectiveness and cost yielded by the Monte Carlo simulations. Green points represent simulations with an incremental cost-effectiveness ratio (ICER) below 5 million yen per QALY gained, and red points represent ICERs above this threshold. The central ellipse indicates the region containing the 95% confidence interval of the simulated results. The light blue area shows the region of dominance and cost-effective results. The proportion of simulation results in this area is 75.61% for panel (**A**) 30–39 years, 81.08% for panel (**B**) 40–49 years, 87.63% for panel (**C**) 50–59 years, and 87.50% for panel (**D**) ≥ 60 years. WTP: willingness-to-pay

Results of the probabilistic sensitivity analysis using Monte Carlo simulation are shown in the cost-effectiveness acceptance curve (Fig. 3) and the incremental cost-effectiveness scatter plot (Fig. 4). For individuals in their 40 s, the combined probability of the early antihypertensive drug administration strategy being both low-cost and effective (dominance probability) and being cost-increasing yet effective with an ICER below ¥5,000,000/QALY gained (cost-effectiveness probability) was 81.08%. Details are shown in Supplementary Table 8.

## Discussion

In this model, using Japanese claims and health checkup data, initiating antihypertensive therapy within 1 year after hypertension is first detected at screening was found to be highly cost-effective compared with initiating treatment at ≥1 year across adults in their 30 s, 40 s, 50 s, and ≥60 s. The early initiation strategy required additional antihypertensive drug costs in the early years, but it substantially reduced the incidence of CVD events over the long term. As a result, ICERs were consistently below the commonly cited WTP threshold of ¥5,000,000 per QALY in Japan. These findings were robust to extensive sensitivity and scenario analyses, including shorter time horizons and alternative assumptions about the duration of additional treatment exposure.

The present results are consistent with accumulating evidence that pharmacological treatment of hypertension is generally cost-effective across a broad range of risk profiles and health system settings. Modeling studies from the United States, Europe, and China have demonstrated that treating hypertension yields ICERs well below commonly accepted thresholds [15, 17, 30]. Furthermore, with the revision of the US hypertension treatment guidelines, the cost-effectiveness of intervening in early-stage hypertension has been demonstrated [16]. For external context, Bress et al. reported annual rates of 1.65 vs. 2.19 per 100 person-years for the composite cardiovascular outcome (intensive vs. standard blood pressure control) in a SPRINT-based model [31], closely matching our cohort’s CVD incidence in similarly aged adults (1.647% at age 40 s, 2.182% at age 50 s). Non-cardiovascular death rates in our cohort were lower (0.182–0.663% vs. 0.78–0.96 per 100 person-years), likely reflecting SPRINT’s older, higher-risk trial population. However, most previous evaluations have focused on whether to treat hypertension or on alternative drug regimens, rather than on when to initiate treatment after screen-detected hypertension. Our previous cohort study demonstrated that delaying antihypertensive therapy by more than 1 year after health checkup detection was associated with increased risks of CVD events. Building upon these clinical findings, the present study extends the evidence by quantifying the economic implications of treatment timing and showing that the benefits of earlier initiation outweigh the additional drug costs over the long term.

In Japan, health checkup programs are widely implemented under the national health insurance system and are a cornerstone of primary prevention of cardiovascular disease. The Japanese Society of Hypertension Guidelines 2025 recommend timely confirmation and management of hypertension detected at screening to prevent progression and complications [32, 33]. The present findings provide quantitative economic support for existing recommendations that emphasize timely management of hypertension detected during routine health checkups. The results indicate that initiating pharmacological therapy within one year of detection is likely to be an efficient use of healthcare resources, including for those in their 30 s and 40 s. These findings suggest that ensuring prompt evaluation and management following elevated blood pressure measurements at screening may help maximize long-term clinical and economic benefits. In practical terms, this includes minimizing delays in follow-up assessment, facilitating timely access to clinical evaluation and diagnostic confirmation, and promoting systems that support appropriate initiation of antihypertensive therapy. From a health system perspective, investments that reduce delays in treatment initiation appear unlikely to impose additional long-term budgetary burdens.

Though early treatment was cost-effective in all age strata, the absolute QALY gains and ICERs varied by age. In younger age groups, early treatment confers benefits over a longer remaining lifespan, allowing more CVD events to be prevented, but it also requires a longer duration of antihypertensive therapy. In older age groups, the shorter remaining life expectancy reduces the period over which benefits accumulate, but baseline CVD risk is higher, so absolute event reductions per treated year can still be substantial.

The age-stratified ICERs fell within a narrow, consistent range of approximately -¥15,000–140,000 per QALY gained. This demonstrates the robustness of the economic benefits of early treatment across different age groups. These findings may inform national screening policies by highlighting the economic advantage of minimizing periods of untreated hypertension after routine health checkups. Furthermore, the present results imply that the initiation of antihypertensive therapy should be encouraged for all adults with screening-detected hypertension, not just the oldest or highest-risk individuals. However, treatment intensity and medication selection should still be tailored to individual clinical characteristics and comorbidities.

### Study limitations

Several limitations should be acknowledged. First, although treatment effects were modeled based on observational data with adjustment for measured confounders, residual confounding (for example, due to lifestyle factors or adherence) cannot be excluded. Patients who initiate treatment early may differ systematically from those who delay treatment in ways not fully captured in claims data. Second, treatment adherence, which is known to affect both clinical outcomes and cost-effectiveness [27], was not explicitly modeled. Third, though the JMDC database covers a wide range of working-age and early elderly populations, it may not be fully representative of the oldest old or those outside employer-based insurance schemes. In addition, indirect costs, such as productivity loss or informal care, which may be particularly relevant in younger working populations, were not included. Including such costs would likely further improve the cost-effectiveness of early initiation by preventing disability and maintaining work capacity. Fourth, the range of TTI is relatively wide in the delayed treatment group, so there may be differences in the impact on CVD events. However, the median delay period was 1067 days, and approximately 80% of patients began treatment within five years. Finally, the structural assumptions, such as not modeling recurrent CVD events separately and excluding explicit end-of-life costs, may have led to some under- or overestimation of long-term costs and QALYs, although sensitivity analyses indicate that these factors are unlikely to reverse the main conclusions.

### Perspective of Asia

While elevated blood pressure increases cardiovascular risk across populations, the relationship between blood pressure and cardiovascular events, particularly stroke, appears disproportionately stronger in Asian populations, where stroke rather than ischemic heart disease predominates [34]. Therefore, further studies are needed to confirm the generalizability of these cost-effectiveness findings outside Asia.

## Conclusions

In this Markov model-based cost-effectiveness analysis using Japanese claims and health checkup data, initiating antihypertensive therapy within 1 year after hypertension is detected at health screening was consistently cost-effective compared with later initiation across adults in their 30 s, 40 s, 50 s, and ≥60 s.

## Supplementary information

Supplementary Information
