# Supplementary material for: Cost-effectiveness analysis of early antihypertensive treatment for hypertension detected at health screening
Source: Hypertens Res. 2026 Aug 19;49(10):2655–65. doi: 10.1038/s41440-026-02776-5 (PMC13634919; doi:10.1038/s41440-026-02776-5)
Supplement: Supplementary file 1 [file 41440_2026_2776_MOESM1_ESM.docx]

**Supplemental Material**

**Table of contents**

Supplementary Table 1. Age-group stratified clinical characteristics of the study population ……………………………..2-7

Supplementary Table 2. Utilities of cardiovascular event components……………………..………………………………….8

Supplementary Table 3. Per-protocol sensitivity analysis…………………..………………………………………………….9

Supplementary Table 4. Poor-prognosis exclusion sensitivity analysis…………………..…………………………………….9

Supplementary Table 5. Scenario analysis……………………………………………………….………………………...10-11

Supplementary Table 6. Scenario analysis……………………………………………………….………………………...12-13

Supplementary Table 7. Scenario analysis……………………………………………………….………………………...14-15

Supplementary Table 8. Monte Carlo incremental cost-effectiveness scatterplot reports………………………………....16-17

Supplementary Materials References……………………………………………………………………………………...18-19

**Supplementary Table 1.** Age-group stratified clinical characteristics of the study population

|  |  | **Time to treatment initiation (TTI)** | |  |  |
| --- | --- | --- | --- | --- | --- |
| **Variable** | **Missing** | **< 1 year** | **≥ 1 years** | ***P*-value** | ***Std diff*** |
| N | | | | | |
| 30-39 years |  | 11847 | 15160 |  |  |
| 40-49 years |  | 80490 | 70702 |  |  |
| 50-59 years |  | 150353 | 59875 |  |  |
| ≥60 years |  | 113735 | 20434 |  |  |
| Median follow-up, years | | | | | |
| 30-39 years |  | 2.44 | 1.63 |  |  |
| 40-49 years |  | 3.12 | 1.83 |  |  |
| 50-59 years |  | 2.87 | 1.65 |  |  |
| ≥60 years |  | 1.94 | 1.24 |  |  |
| Age, years | | | | | |
| 30-39 years | 0 | 36.49 (2.58) | 36.37 (2.60) | <0.001 | 0.048 |
| 40-49 years | 0 | 45.31 (2.84) | 44.65 (2.90) | <0.001 | 0.229 |
| 50-59 years | 0 | 54.67 (2.84) | 53.95 (2.80) | <0.001 | 0.257 |
| ≥60 years | 0 | 64.17 (3.64) | 63.21 (3.06) | <0.001 | 0.287 |
| Male sex, n (%) | | | | | |
| 30-39 years | 0 | 9218 (77.81) | 11549 (76.18) | 0.002 | 0.039 |
| 40-49 years | 0 | 57943 (71.99) | 52004 (73.55) | <0.001 | 0.035 |
| 50-59 years | 0 | 103441 (68.80) | 43782 (73.12) | <0.001 | 0.095 |
| ≥60 years | 0 | 75072 (66.01) | 13670 (66.90) | 0.013 | 0.019 |
| Body mass index, kg/m^2^ | | | | | |
| 30-39 years | 13 | 28.18 (5.99) | 26.21 (5.02) | <0.001 | 0.358 |
| 40-49 years | 71 | 26.85 (4.98) | 25.27 (4.17) | <0.001 | 0.343 |
| 50-59 years | 89 | 25.43 (4.14) | 24.22 (3.50) | <0.001 | 0.315 |
| ≥60 years | 54 | 24.42 (3.49) | 23.47 (3.16) | <0.001 | 0.287 |
| Overweight, n (%) | | | | | |
| 30-39 years | 13 | 8003 (67.55) | 8322 (54.89) | <0.001 | 0.262 |
| 40-49 years | 71 | 49094 (60.99) | 33662 (47.61) | <0.001 | 0.271 |
| 50-59 years | 89 | 75086 (49.94) | 22097 (36.91) | <0.001 | 0.266 |
| ≥60 years | 54 | 45198 (39.74) | 5865 (28.70) | <0.001 | 0.234 |
| Waist circumference, cm | | | | | |
| 30-39 years | 3690 | 93.44 (14.28) | 88.80 (12.34) | <0.001 | 0.347 |
| 40-49 years | 1632 | 90.73 (12.18) | 87.02 (10.48) | <0.001 | 0.327 |
| 50-59 years | 1694 | 88.40 (10.44) | 85.44 (9.18) | <0.001 | 0.302 |
| ≥60 years | 693 | 86.73 (9.16) | 84.31 (8.60) | <0.001 | 0.273 |
| Visceral obesity, n (%) | | | | | |
| 30-39 years | 3690 | 7315 (61.75) | 7063 (46.59) | <0.001 | 0.319 |
| 40-49 years | 1632 | 49827 (61.90) | 35533 (50.26) | <0.001 | 0.237 |
| 50-59 years | 1694 | 83739 (55.69) | 27002 (45.10) | <0.001 | 0.214 |
| ≥60 years | 693 | 56983 (50.10) | 8066 (39.47) | <0.001 | 0.217 |
| Systolic blood pressure, mm Hg | | | | | |
| 30-39 years | 0 | 141.51 (16.65) | 137.47 (13.59) | <0.001 | 0.266 |
| 40-49 years | 0 | 139.05 (15.90) | 136.93 (13.63) | <0.001 | 0.143 |
| 50-59 years | 0 | 137.88 (14.81) | 138.59 (14.26) | <0.001 | 0.049 |
| ≥60 years | 0 | 140.02 (14.19) | 141.58 (14.63) | <0.001 | 0.108 |
| Diastolic blood pressure, mm Hg | | | | | |
| 30-39 years | 0 | 91.30 (11.96) | 88.50 (9.69) | <0.001 | 0.257 |
| 40-49 years | 0 | 90.05 (10.47) | 88.76 (9.16) | <0.001 | 0.131 |
| 50-59 years | 0 | 87.42 (9.14) | 88.27 (9.02) | <0.001 | 0.093 |
| ≥60 years | 0 | 83.98 (9.21) | 85.83 (9.28) | <0.001 | 0.2 |
| Stage 1 hypertension, n (%) | | | | | |
| 30-39 years | 0 | 5027 (42.43) | 7864 (51.87) | <0.001 | 0.19 |
| 40-49 years | 0 | 38003 (47.21) | 36356 (51.42) | <0.001 | 0.084 |
| 50-59 years | 0 | 79196 (52.67) | 28998 (48.43) | <0.001 | 0.085 |
| ≥60 years | 0 | 59079 (51.94) | 9265 (45.34) | <0.001 | 0.132 |
| Stage 2 hypertension, n (%) | | | | | |
| 30-39 years | 0 | 6820 (57.57) | 7296 (48.13) | <0.001 | 0.19 |
| 40-49 years | 0 | 42487 (52.79) | 34346 (48.58) | <0.001 | 0.084 |
| 50-59 years | 0 | 71157 (47.33) | 30877 (51.57) | <0.001 | 0.085 |
| ≥60 years | 0 | 54656 (48.06) | 11169 (54.66) | <0.001 | 0.132 |
| Triglycerides, mg/dL | | | | | |
| 30-39 years | 901 | 158.28 (129.16) | 146.43 (125.70) | <0.001 | 0.093 |
| 40-49 years | 339 | 150.92 (122.99) | 141.27 (117.26) | <0.001 | 0.08 |
| 50-59 years | 368 | 140.80 (109.33) | 134.74 (107.14) | <0.001 | 0.056 |
| ≥60 years | 268 | 128.09 (87.75) | 123.95 (86.24) | <0.001 | 0.048 |
| HDL cholesterol, mg/dL | | | | | |
| 30-39 years | 878 | 54.63 (14.87) | 56.14 (15.11) | <0.001 | 0.101 |
| 40-49 years | 254 | 57.76 (16.07) | 58.79 (15.98) | <0.001 | 0.065 |
| 50-59 years | 301 | 61.19 (16.94) | 61.93 (17.01) | <0.001 | 0.044 |
| ≥60 years | 249 | 62.30 (16.76) | 63.31 (16.95) | <0.001 | 0.06 |
| LDL cholesterol, mg/dL | | | | | |
| 30-39 years | 960 | 125.34 (32.56) | 125.98 (33.18) | 0.116 | 0.02 |
| 40-49 years | 361 | 124.77 (31.35) | 127.93 (32.21) | <0.001 | 0.099 |
| 50-59 years | 421 | 123.96 (30.53) | 129.91 (32.06) | <0.001 | 0.19 |
| ≥60 years | 282 | 121.49 (29.48) | 129.51 (31.47) | <0.001 | 0.263 |
| AST, U/L | | | | | |
| 30-39 years | 841 | 29.86 (20.28) | 26.73 (16.41) | <0.001 | 0.17 |
| 40-49 years | 238 | 27.40 (16.93) | 24.82 (13.64) | <0.001 | 0.168 |
| 50-59 years | 286 | 26.45 (14.80) | 24.60 (12.74) | <0.001 | 0.134 |
| ≥60 years | 237 | 25.66 (13.33) | 24.45 (11.13) | <0.001 | 0.098 |
| ALT, U/L | | | | | |
| 30-39 years | 841 | 43.70 (36.88) | 37.34 (32.14) | <0.001 | 0.184 |
| 40-49 years | 236 | 35.11 (27.04) | 30.44 (23.53) | <0.001 | 0.184 |
| 50-59 years | 282 | 29.53 (21.05) | 26.26 (18.62) | <0.001 | 0.165 |
| ≥60 years | 226 | 25.34 (17.39) | 23.13 (14.62) | <0.001 | 0.138 |
| γ-glutamyl transpeptidase, IU/L | | | | | |
| 30-39 years | 866 | 60.71 (67.11) | 51.75 (53.02) | <0.001 | 0.148 |
| 40-49 years | 308 | 59.32 (66.16) | 52.86 (58.66) | <0.001 | 0.103 |
| 50-59 years | 404 | 58.00 (67.01) | 52.63 (59.54) | <0.001 | 0.085 |
| ≥60 years | 282 | 48.95 (56.30) | 45.78 (54.07) | <0.001 | 0.057 |
| Fasting blood glucose, mg/dL | | | | | |
| 30-39 years | 5747 | 102.25 (29.53) | 96.18 (22.37) | <0.001 | 0.232 |
| 40-49 years | 22490 | 104.33 (27.41) | 98.12 (22.15) | <0.001 | 0.249 |
| 50-59 years | 5747 | 106.58 (25.56) | 101.19 (22.28) | <0.001 | 0.225 |
| ≥60 years | 18916 | 107.80 (23.90) | 103.49 (21.49) | <0.001 | 0.189 |
| HbA_1c_, % | | | | | |
| 30-39 years | 4865 | 5.75 (1.05) | 5.53 (0.83) | <0.001 | 0.233 |
| 40-49 years | 16399 | 5.80 (0.97) | 5.60 (0.79) | <0.001 | 0.228 |
| 50-59 years | 21596 | 5.87 (0.87) | 5.70 (0.77) | <0.001 | 0.204 |
| ≥60 years | 16208 | 5.91 (0.76) | 5.79 (0.72) | <0.001 | 0.165 |
| Hemoglobin, g/dL | | | | | |
| 30-39 years | 6031 | 15.15 (1.59) | 15.07 (1.55) | 0.001 | 0.047 |
| 40-49 years | 43251 | 14.85 (1.62) | 14.82 (1.67) | 0.001 | 0.02 |
| 50-59 years | 62213 | 14.74 (1.35) | 14.79 (1.40) | <0.001 | 0.038 |
| ≥60 years | 41995 | 14.47 (1.30) | 14.54 (1.28) | <0.001 | 0.054 |
| Cigarette smoking, n (%) | | | | | |
| 30-39 years | 79 | 3623 (30.58) | 5557 (36.66) | <0.001 | 0.131 |
| 40-49 years | 146 | 23581 (29.30) | 24273 (34.33) | <0.001 | 0.108 |
| 50-59 years | 27 | 38993 (25.93) | 19322 (32.27) | <0.001 | 0.14 |
| ≥60 years | 27 | 22338 (19.64) | 5165 (25.28) | <0.001 | 0.136 |
| Diabetes, n (%) | | | | | |
| 30-39 years | 5750 | 942 (7.95) | 475 (3.13) | <0.001 | 0.215 |
| 40-49 years | 22526 | 7695 (9.56) | 2999 (4.24) | <0.001 | 0.212 |
| 50-59 years | 1437 | 17414 (11.58) | 3606 (6.02) | <0.001 | 0.197 |
| ≥60 years | 1437 | 14285 (12.56) | 1565 (7.66) | <0.001 | 0.163 |
| Dyslipidemia, n (%) | | | | | |
| 30-39 years | 1003 | 6528 (55.10) | 7657 (50.51) | <0.001 | 0.092 |
| 40-49 years | 515 | 43425 (53.95) | 37234 (52.66) | <0.001 | 0.027 |
| 50-59 years | 666 | 76568 (50.93) | 31906 (53.29) | <0.001 | 0.048 |
| ≥60 years | 666 | 51376 (45.17) | 10357 (50.69) | <0.001 | 0.112 |
| Antihypertensive agents, n (%) | | | | | |
| Calcium channel blockers | | | | | |
| 30-39 years | 0 | 9026 (76.19) | 11380 (75.07) | 0.034 | 0.026 |
| 40-49 years | 0 | 63885 (79.37) | 54171 (76.62) | <0.001 | 0.066 |
| 50-59 years | 0 | 118725 (78.96) | 45130 (75.37) | <0.001 | 0.086 |
| ≥60 years | 0 | 89192 (78.42) | 15208 (74.42) | <0.001 | 0.094 |
| Beta blockers | | | | | |
| 30-39 years | 0 | 2240 (18.91) | 2390 (15.77) | <0.001 | 0.083 |
| 40-49 years | 0 | 13801 (17.15) | 9791 (13.85) | <0.001 | 0.091 |
| 50-59 years | 0 | 22915 (15.24) | 7998 (13.36) | <0.001 | 0.054 |
| ≥60 years | 0 | 15601 (13.72) | 2596 (12.70) | <0.001 | 0.030 |
| Angiotensin-converting enzyme inhibitors | | | | | |
| 30-39 years | 0 | 638 (5.39) | 777 (5.13) | 0.355 | 0.012 |
| 40-49 years | 0 | 4184 (5.20) | 3251 (4.60) | <0.001 | 0.028 |
| 50-59 years | 0 | 7651 (5.09) | 2692 (4.50) | <0.001 | 0.028 |
| ≥60 years | 0 | 6160 (5.42) | 953 (4.66) | <0.001 | 0.034 |
| Angiotensin II receptor blockers | | | | | |
| 30-39 years | 0 | 8123 (68.57) | 8869 (58.50) | <0.001 | 0.21 |
| 40-49 years | 0 | 58781 (73.03) | 43716 (61.83) | <0.001 | 0.241 |
| 50-59 years | 0 | 107358 (71.40) | 35114 (58.65) | <0.001 | 0.27 |
| ≥60 years | 0 | 76283 (67.07) | 10930 (53.49) | <0.001 | 0.280 |
| Direct renin inhibitors | | | | | |
| 30-39 years | 0 | 20 (0.17) | 14 (0.09) | 0.113 | 0.021 |
| 40-49 years | 0 | 201 (0.25) | 48 (0.07) | <0.001 | 0.046 |
| 50-59 years | 0 | 259 (0.17) | 37 (0.06) | <0.001 | 0.032 |
| ≥60 years | 0 | 180 (0.16) | 11 (0.05) | <0.001 | 0.032 |
| Angiotensin receptor neprilysin inhibitor | | | | | |
| 30-39 years | 0 | 4 (0.03) | 1 (0.01) | 0.239 | 0.019 |
| 40-49 years | 0 | 13 (0.02) | 18 (0.03) | 0.28 | 0.006 |
| 50-59 years | 0 | 18 (0.01) | 12 (0.02) | 0.232 | 0.006 |
| ≥60 years | 0 | 8 (0.01) | 3 (0.01) | 0.498 | 0.007 |
| Antihypertensive diuretics | | | | | |
| 30-39 years | 0 | 1331 (11.23) | 1209 (7.97) | <0.001 | 0.111 |
| 40-49 years | 0 | 10521 (13.07) | 5502 (7.78) | <0.001 | 0.174 |
| 50-59 years | 0 | 17913 (11.91) | 4103 (6.85) | <0.001 | 0.174 |
| ≥60 years | 0 | 11558 (10.16) | 1143 (5.59) | <0.001 | 0.170 |
| Alpha-adrenergic agents | | | | | |
| 30-39 years | 0 | 10 (0.08) | 8 (0.05) | 0.446 | 0.012 |
| 40-49 years | 0 | 53 (0.07) | 25 (0.04) | 0.013 | 0.014 |
| 50-59 years | 0 | 48 (0.03) | 24 (0.04) | 0.434 | 0.004 |
| ≥60 years | 0 | 17 (0.01) | 2 (0.01) | 0.802 | 0.005 |
| Alpha blockers | | | | | |
| 30-39 years | 0 | 537 (4.53) | 457 (3.01) | <0.001 | 0.08 |
| 40-49 years | 0 | 3556 (4.42) | 2011 (2.84) | <0.001 | 0.084 |
| 50-59 years | 0 | 6073 (4.04) | 1665 (2.78) | <0.001 | 0.069 |
| ≥60 years | 0 | 4249 (3.74) | 557 (2.73) | <0.001 | 0.057 |

Categorical variables are expressed as frequency (proportion) unless otherwise indicated. Continuous variables are shown as mean (standard deviation).

**Supplementary Table 2.** Utilities of cardiovascular event components

| Components | Utility values (range for sensitivity analysis) | References |
| --- | --- | --- |
| **Acute phase** | | |
| Acute coronary syndrome | 0.74 (0.67 – 0.80) | [1, 2] |
| Heart failure | 0.61 (0.56 – 0.71) | [1, 3-6] |
| Stroke | 0.42 (0.52 – 0.70) | [1, 2, 7] |
| **Chronic phase** | | |
| Acute coronary syndrome | 0.78 (0.73 - 0.87) | [1, 2, 8, 9] |
| Heart failure | 0.67 (0.51 – 0.81) | [1, 3, 10-26] |
| Stroke | 0.51 (0.52 – 0.70) | [1, 2, 7, 27, 28] |

**Supplementary Table 3**. Per-protocol sensitivity analysis: parameters excluding individuals who discontinued antihypertensive treatment within 1 year

|  | **Input (range for sensitivity analysis)** | | | |
| --- | --- | --- | --- | --- |
| Parameters | Age: 30-39 y | Age: 40-49 y | Age: 50-59 y | Age: ≥60 y |
| Probabilities | | | | |
| Annual incidence rates of CVD, % | 1.310 | 1.635 | 2.163 | 2.582 |
| Annual mortality rates by CVD, % | 5.110 | 5.401 | 5.122 | 4.238 |
| Annual mortality rates by non-CVD, % | 0.113 | 0.286 | 0.409 | 0.542 |
| Effectiveness of intervention (risk difference) | | | | |
| Composite CVD event | 0.757 | 0.950 | 1.440 | 1.632 |
| CVD death | 1.486 | 2.105 | 0.886 | 0.609 |
| Non-CVD death | 0.014 | 0.207 | 0.289 | 0.371 |

**Supplementary Table 4**. Poor-prognosis exclusion sensitivity analysis: parameters excluding individuals with malignancy

|  | **Input (range for sensitivity analysis)** | | | |
| --- | --- | --- | --- | --- |
| Parameters | Age: 30-39 y | Age: 40-49 y | Age: 50-59 y | Age: ≥60 y |
| Probabilities | | | | |
| Annual incidence rates of CVD, % | 1.240 | 1.646 | 2.181 | 2.587 |
| Annual mortality rates by CVD, % | 4.904 | 4.897 | 5.152 | 4.153 |
| Annual mortality rates by non-CVD, % | 0.182 | 0.276 | 0.453 | 0.659 |
| Effectiveness of intervention (risk difference) | | | | |
| Composite CVD event | 0.639 | 0.898 | 1.396 | 1.581 |
| CVD death | 1.095 | 1.161 | 0.690 | 0.174 |
| Non-CVD death | 0.092 | 0.188 | 0.332 | 0.460 |

**Supplementary Table 5.** Scenario analysis: results of scenario analysis conducted assuming an initial incremental cost of 0.5 years

|  | Age: 30-39 y | | Age: 40-49 y | | Age: 50-59 y | | Age: ≥60 y | |
| --- | --- | --- | --- | --- | --- | --- | --- | --- |
|  | TTI < 1 year | TTI ≥ 1 year | TTI < 1 year | TTI ≥ 1 year | TTI < 1 year | TTI ≥ 1 year | TTI < 1 year | TTI ≥ 1 year |
| 20-year time horizon (main) | | | | | | | | |
| Costs | ¥3,893,620  ($26,487) | ¥3,918,410  ($26,656) | ¥3,935,316  ($26,771) | ¥3,976,709  ($27,052) | ¥4,334,658  ($29,487) | ¥4,411,692  ($30,012) | ¥5,344,778  ($36,359) | ¥5,306,798  ($36,101) |
| QALYs | 13.61 | 13.16 | 13.52 | 12.85 | 12.99 | 12.05 | 11.50 | 10.66 |
| ICER | -¥55,200 ($376)/QALY | | -¥61,859 ($421)/QALY | | -¥81,815 (-$557)/QALY | | ¥45,318 ($308)/QALY | |
| 30-year time horizon | | | | | | | | |
| Costs | ¥5,290,274  ($35,988) | ¥5,291,741  ($35,998) | ¥5,350,714  ($36,399) | ¥5,341,409  ($36,336) | ¥5,863,725  ($39,889) | ¥5,847,963  ($39,782) |  |  |
| QALYs | 18.30 | 17.37 | 18.12 | 16.76 | 17.37 | 15.48 |  |  |
| ICER | -¥1,561 ($11)/QALY | | ¥9,305 ($63)/QALY | | ¥8,323 ($57)/QALY | |  | |
| 40-year time horizon | | | | | | | | |
| Costs | ¥6,394,655  ($43,501) | ¥6,324,831  ($43,026) | ¥6,465,337  ($43,982) | ¥6,339,036  ($43,123) |  |  |  |  |
| QALYs | 21.95 | 20.44 | 21.66 | 19.50 |  |  |  |  |
| ICER | ¥46,180 ($314)/QALY | | ¥58,565 ($398)/QALY | |  | |  | |
| 50-year time horizon | | | | | | | | |
| Costs | ¥7,258,494  ($49,378) | ¥7,086,993  ($38,211) |  |  |  |  |  |  |
| QALYs | 24.77 | 22.67 |  |  |  |  |  |  |
| ICER | ¥81,541 ($555)/QALY | |  | |  | |  | |

**Supplementary Table 6.** Scenario analysis: results of sensitivity analysis excluding individuals who discontinued antihypertensive treatment within 1 year of initiation

|  | Age: 30-39 y | | Age: 40-49 y | | Age: 50-59 y | | Age: ≥60 y | |
| --- | --- | --- | --- | --- | --- | --- | --- | --- |
|  | TTI < 1 year | TTI ≥ 1 year | TTI < 1 year | TTI ≥ 1 year | TTI < 1 year | TTI ≥ 1 year | TTI < 1 year | TTI ≥ 1 year |
| 20-year time horizon (main) | | | | | | | | |
| Costs | ¥3,945,430  ($26,840) | ¥3,952,749  ($26,889) | ¥4,000,391  ($27,214) | ¥3,957,937  ($26,925) | ¥4,422,714  ($30,086) | ¥4,416,258  ($30,043) | ¥5,452,195  ($37,090) | ¥5,357,522  ($36,446) |
| QALYs | 13.64 | 13.20 | 13.61 | 12.84 | 13.09 | 12.09 | 11.58 | 10.76 |
| ICER | -¥16,789 ($114)/QALY | | ¥54,769 ($373)/QALY | | ¥6,458 ($44)/QALY | | ¥115,767 ($788)/QALY | |
| 30-year time horizon | | | | | | | | |
| Costs | ¥5,351,069  ($36,402) | ¥5,346,240  ($36,369) | ¥5,440,567  ($37,011) | ¥5,300,377  ($36,057) | ¥6,001,276  ($40,825) | ¥5,855,207  ($39,831) |  |  |
| QALYs | 18.38 | 17.44 | 18.32 | 16.72 | 17.59 | 15.55 |  |  |
| ICER | ¥5,121 ($35)/QALY | | ¥87,681 ($596)/QALY | | ¥71,410 ($486)/QALY | |  | |
| 40-year time horizon | | | | | | | | |
| Costs | ¥6,471,504  ($44,034) | ¥6,395,709  ($43,508) | ¥6,590,384  ($44,833) | ¥6,274,201  ($42,682) |  |  |  |  |
| QALYs | 22.08 | 20.53 | 21.97 | 19.44 |  |  |  |  |
| ICER | ¥48,938 ($333)/QALY | | ¥124,739 ($849)/QALY | |  | |  | |
| 50-year time horizon | | | | | | | | |
| Costs | ¥7,355,327  ($50,036) | ¥7,170,309  ($48,778) |  |  |  |  |  |  |
| QALYs | 24.96 | 22.77 |  |  |  |  |  |  |
| ICER | ¥84,712 ($576)/QALY | |  | |  | |  | |

**Supplementary Table 7.** Scenario analysis: results of sensitivity analysis excluding individuals with malignancy

|  | Age: 30-39 y | | Age: 40-49 y | | Age: 50-59 y | | Age: ≥60 y | |
| --- | --- | --- | --- | --- | --- | --- | --- | --- |
|  | TTI < 1 year | TTI ≥ 1 year | TTI < 1 year | TTI ≥ 1 year | TTI < 1 year | TTI ≥ 1 year | TTI < 1 year | TTI ≥ 1 year |
| 20-year time horizon (main) | | | | | | | | |
| Costs | ¥3,957,076  ($26,919) | ¥3,922,952  ($26,687) | ¥4,003,906  ($27,237) | ¥3,983,572  ($27,099) | ¥4,410,994  ($30,007) | ¥4,417,276  ($30,049) | ¥5,438,582  ($36,997) | ¥5,315,129  ($36,157) |
| QALYs | 13.62 | 13.17 | 13.56 | 12.87 | 13.03 | 12.06 | 11.52 | 10.67 |
| ICER | ¥74,790 ($509)/QALY | | ¥29,621 ($202)/QALY | | -¥6,488 (-$44)/QALY | | ¥144,568 ($983)/QALY | |
| 30-year time horizon | | | | | | | | |
| Costs | ¥5,365,567  ($36,500) | ¥5,302,343  ($36,070) | ¥5,433,571  ($36,963) | ¥5,355,104  ($36,429) | ¥5,954,881  ($40,509) | ¥5,860,255  ($39,866) |  |  |
| QALYs | 18.34 | 17.38 | 18.20 | 16.79 | 17.44 | 15.50 |  |  |
| ICER | ¥65,977 ($449)/QALY | | ¥55,848 ($380)/QALY | | ¥48,636 ($331)/QALY | |  | |
| 40-year time horizon | | | | | | | | |
| Costs | ¥6,485,020  ($44,116) | ¥6,342,312  ($43,145) | ¥6,565,006  ($44,660) | ¥6,359,915  ($43,265) |  |  |  |  |
| QALYs | 22.01 | 20.46 | 21.78 | 19.56 |  |  |  |  |
| ICER | ¥92,331 ($628)/QALY | | ¥92,426 ($629)/QALY | |  | |  | |
| 50-year time horizon | | | | | | | | |
| Costs | ¥7,364,876  ($50,101) | ¥7,111,072  ($48,375) |  |  |  |  |  |  |
| QALYs | 24.85 | 22.70 |  |  |  |  |  |  |
| ICER | ¥117,844 ($802)/QALY | |  | |  | |  | |

**Supplementary Table 8.** Monte Carlo incremental cost-effectiveness scatterplot reports

| Age: 30-39y | | | | | | |
| --- | --- | --- | --- | --- | --- | --- |
| **Quadrant** | **IC** | **IE** | **ICER** | **Frequency** | | **Proportion** |
| IV | IC<0 | IE>0 | Superior | 44533 | | 0.44533 |
| Ia | IC>0 | IE>0 | ICER<5000000 | 31078 | | 0.31078 |
| IIIb | IC<0 | IE<0 | ICER>5000000 | 465 | | 0.00465 |
| Ib | IC>0 | IE>0 | ICER>5000000 | 2299 | | 0.02299 |
| IIIa | IC<0 | IE<0 | ICER<5000000 | 4068 | | 0.04068 |
| II | IC>0 | IE<0 | Inferior | 17557 | | 0.17557 |
| Age: 40-49y | | | | | | |
| **Quadrant** | **IC** | **IE** | **ICER** | | **Frequency** | **Proportion** |
| IV | IC<0 | IE>0 | Superior | | 47663 | 0.47663 |
| Ia | IC>0 | IE>0 | ICER<5000000 | | 33412 | 0.33412 |
| IIIb | IC<0 | IE<0 | ICER>5000000 | | 309 | 0.00309 |
| Ib | IC>0 | IE>0 | ICER>5000000 | | 1460 | 0.0146 |
| IIIa | IC<0 | IE<0 | ICER<5000000 | | 3767 | 0.03767 |
| II | IC>0 | IE<0 | Inferior | | 13389 | 0.13389 |
| Age: 50-59y |  |  |  | |  |  |
| **Quadrant** | **IC** | **IE** | **ICER** | | **Frequency** | **Proportion** |
| IV | IC<0 | IE>0 | Superior | | 50171 | 0.50171 |
| Ia | IC>0 | IE>0 | ICER<5000000 | | 37454 | 0.37454 |
| IIIb | IC<0 | IE<0 | ICER>5000000 | | 251 | 0.00251 |
| Ib | IC>0 | IE>0 | ICER>5000000 | | 993 | 0.00993 |
| IIIa | IC<0 | IE<0 | ICER<5000000 | | 4076 | 0.04076 |
| II | IC>0 | IE<0 | Inferior | | 7055 | 0.07055 |
| Age: ≥60y |  |  |  | |  |  |
| **Quadrant** | **IC** | **IE** | **ICER** | | **Frequency** | **Proportion** |
| IV | IC<0 | IE>0 | Superior | | 35613 | 0.35613 |
| Ia | IC>0 | IE>0 | ICER<5000000 | | 51887 | 0.51887 |
| IIIb | IC<0 | IE<0 | ICER>5000000 | | 474 | 0.00474 |
| Ib | IC>0 | IE>0 | ICER>5000000 | | 1193 | 0.01193 |
| IIIa | IC<0 | IE<0 | ICER<5000000 | | 6130 | 0.0613 |
| II | IC>0 | IE<0 | Inferior | | 4703 | 0.04703 |

A total of 100,000 Monte Carlo simulations were performed. IC: incremental cost; IE: incremental effectiveness; ICER: incremental cost-effectiveness ratio. The following diagram illustrates the meaning of the quadrants. The combined area of Quadrants Ia and IV (light blue field) shows that the treatment strategy being evaluated is superior.

**Supplementary Materials References**

1.	Matza LS, Stewart KD, Gandra SR, Delio PR, Fenster BE, Davies EW, et al. Acute and chronic impact of cardiovascular events on health state utilities. BMC Health Serv Res. 2015;15:173.

2.	Kodera S, Morita H, Kiyosue A, Ando J, Takura T, Komuro I. Cost-Effectiveness of PCSK9 Inhibitor Plus Statin in Patients With Triple-Vessel Coronary Artery Disease in Japan. Circ J. 2018;82:2602-8.

3.	Ambrosy AP, Hernandez AF, Armstrong PW, Butler J, Dunning A, Ezekowitz JA, et al. The clinical course of health status and association with outcomes in patients hospitalized for heart failure: insights from ASCEND-HF. Eur J Heart Fail. 2016;18:306-13.

4.	Hansson E, Ekman I, Swedberg K, Wolf A, Dudas K, Ehlers L, Olsson LE. Person-centred care for patients with chronic heart failure - a cost-utility analysis. Eur J Cardiovasc Nurs. 2016;15:276-84.

5.	Maru S, Byrnes JM, Carrington MJ, Stewart S, Scuffham PA. Long-term cost-effectiveness of home versus clinic-based management of chronic heart failure: the WHICH? study. J Med Econ. 2017;20:318-27.

6.	Van Spall HGC, Lee SF, Xie F, Oz UE, Perez R, Mitoff PR, et al. Effect of Patient-Centered Transitional Care Services on Clinical Outcomes in Patients Hospitalized for Heart Failure: The PACT-HF Randomized Clinical Trial. Jama. 2019;321:753-61.

7.	Zhou J, Wei Q, Hu H, Liu W, Guan X, Ma A, Wang L. A systematic review and meta-analysis of health utility values among patients with ischemic stroke. Front Neurol. 2023;14:1219679.

8.	Timóteo AT, Dias SS, Rodrigues AM, Gregório MJ, Sousa RD, Canhão H. Quality of life in adults living in the community with previous self-reported myocardial infarction. Rev Port Cardiol (Engl Ed). 2020;39:367-73.

9.	Pocock S, Brieger DB, Owen R, Chen J, Cohen MG, Goodman S, et al. Health-related quality of life 1-3 years post-myocardial infarction: its impact on prognosis. Open Heart. 2021;8.

10.	Di Tanna GL, Urbich M, Wirtz HS, Potrata B, Heisen M, Bennison C, et al. Health State Utilities of Patients with Heart Failure: A Systematic Literature Review. Pharmacoeconomics. 2021;39:211-29.

11.	Seo M, Watanabe T, Yamada T, Yano M, Hayashi T, Nakagawa A, et al. The clinical relevance of quality of life in heart failure patients with preserved ejection fraction. ESC Heart Fail. 2023;10:995-1002.

12.	Andreae C, Strömberg A, Chung ML, Hjelm C, Årestedt K. Depressive Symptoms Moderate the Association Between Appetite and Health Status in Patients With Heart Failure. J Cardiovasc Nurs. 2018;33:E15-e20.

13.	Austin J, Williams R, Ross L, Moseley L, Hutchison S. Randomised controlled trial of cardiac rehabilitation in elderly patients with heart failure. Eur J Heart Fail. 2005;7:411-7.

14.	Berg J, Lindgren P, Mejhert M, Edner M, Dahlström U, Kahan T. Determinants of Utility Based on the EuroQol Five-Dimensional Questionnaire in Patients with Chronic Heart Failure and Their Change Over Time: Results from the Swedish Heart Failure Registry. Value Health. 2015;18:439-48.

15.	de Rivas B, Permanyer-Miralda G, Brotons C, Aznar J, Sobreviela E. Health-related quality of life in unselected outpatients with heart failure across Spain in two different health care levels. Magnitude and determinants of impairment: the INCA study. Qual Life Res. 2008;17:1229-38.

16.	Comin-Colet J, Lainscak M, Dickstein K, Filippatos GS, Johnson P, Lüscher TF, et al. The effect of intravenous ferric carboxymaltose on health-related quality of life in patients with chronic heart failure and iron deficiency: a subanalysis of the FAIR-HF study. Eur Heart J. 2013;34:30-8.

17.	Filippatos G, Maggioni AP, Lam CSP, Pieske-Kraigher E, Butler J, Spertus J, et al. Patient-reported outcomes in the SOluble guanylate Cyclase stimulatoR in heArT failurE patientS with PRESERVED ejection fraction (SOCRATES-PRESERVED) study. Eur J Heart Fail. 2017;19:782-91.

18.	Hebert PL, Sisk JE, Wang JJ, Tuzzio L, Casabianca JM, Chassin MR, et al. Cost-effectiveness of nurse-led disease management for heart failure in an ethnically diverse urban community. Ann Intern Med. 2008;149:540-8.

19.	Iqbal J, Francis L, Reid J, Murray S, Denvir M. Quality of life in patients with chronic heart failure and their carers: a 3-year follow-up study assessing hospitalization and mortality. Eur J Heart Fail. 2010;12:1002-8.

20.	Jolly K, Taylor RS, Lip GY, Davies M, Davis R, Mant J, et al. A randomized trial of the addition of home-based exercise to specialist heart failure nurse care: the Birmingham Rehabilitation Uptake Maximisation study for patients with Congestive Heart Failure (BRUM-CHF) study. Eur J Heart Fail. 2009;11:205-13.

21.	Kraai IH, Vermeulen KM, Luttik ML, Hoekstra T, Jaarsma T, Hillege HL. Preferences of heart failure patients in daily clinical practice: quality of life or longevity? Eur J Heart Fail. 2013;15:1113-21.

22.	Li Y, Neilson MP, Whellan DJ, Schulman KA, Levy WC, Reed SD. Associations between Seattle Heart Failure Model scores and health utilities: findings from HF-ACTION. J Card Fail. 2013;19:311-6.

23.	Peters M, Crocker H, Jenkinson C, Doll H, Fitzpatrick R. The routine collection of patient-reported outcome measures (PROMs) for long-term conditions in primary care: a cohort survey. BMJ Open. 2014;4:e003968.

24.	Pisa G, Eichmann F, Hupfer S. Assessing patient preferences in heart failure using conjoint methodology. Patient Prefer Adherence. 2015;9:1233-41.

25.	Wootton R, Gramotnev H, Hailey D. A randomized controlled trial of telephone-supported care coordination in patients with congestive heart failure. J Telemed Telecare. 2009;15:182-6.

26.	Zugck C, Martinka P, Stöckl G. Ivabradine treatment in a chronic heart failure patient cohort: symptom reduction and improvement in quality of life in clinical practice. Adv Ther. 2014;31:961-74.

27.	Luengo-Fernandez R, Gray AM, Bull L, Welch S, Cuthbertson F, Rothwell PM. Quality of life after TIA and stroke: ten-year results of the Oxford Vascular Study. Neurology. 2013;81:1588-95.

28.	Hattori N, Hirayama T, Katayama Y. Medical care for chronic-phase stroke in Japan. Neurol Med Chir (Tokyo). 2012;52:175-80.
